# Circulating Superoxide Dismutase Concentrations in Obstructive Sleep Apnoea (OSA): A Systematic Review and Meta-Analysis

**DOI:** 10.3390/antiox10111764

**Published:** 2021-11-04

**Authors:** Maria Carmina Pau, Arduino Aleksander Mangoni, Elisabetta Zinellu, Gianfranco Pintus, Ciriaco Carru, Alessandro Giuseppe Fois, Pietro Pirina, Angelo Zinellu

**Affiliations:** 1 Department of Medical, Surgical and Experimental Sciences, University of Sassari, 07100 Sassari, Italy; agfois@uniss.it (A.G.F.); pirina@uniss.it (P.P.); 2 Department of Clinical Pharmacology, Flinders University and Flinders Medical Centre, College of Medicine and Public Health, Bedford Park, SA 5042, Australia; arduino.mangoni@flinders.edu.au; 3 Clinical and Interventional Pneumology, University Hospital Sassari (AOU), Viale San Pietro, 07100 Sassari, Italy; elisabetta.zinellu@aousassari.it; 4 Department of Biomedical Sciences, University of Sassari, Viale San Pietro, 07100 Sassari, Italy; gpintus@uniss.it (G.P.); carru@uniss.it (C.C.); azinellu@uniss.it (A.Z.); 5 Department of Medical Laboratory Sciences, Sharjah Institute for Medical Research, College of Health Sciences, University of Sharjah, Sharjah P.O. Box 27272, United Arab Emirates; 6 Quality Control Unit, University Hospital of Sassari (AOU), Viale San Pietro, 07100 Sassari, Italy

**Keywords:** obstructive sleep apnoea, oxidative stress, superoxide dismutase

## Abstract

Obstructive Sleep Apnoea (OSA) is characterized by a pro-oxidant state that results from the recurrent hypoxia-reoxygenation cycles. Superoxide dismutase (SOD), a key antioxidant enzyme involved in the detoxification of superoxide radicals, could represent a reliable marker to monitor the antioxidant defences in OSA. In order to capture and critically appraise the available evidence, we performed a systematic review and meta-analysis of studies reporting SOD concentrations in OSA patients and non-OSA controls in the electronic databases Pubmed, Web of Science, Scopus and Google Scholar. In total, 13 studies in 847 OSA patients and 438 non-OSA controls were included in the meta-analysis. Blood SOD concentrations were significantly lower in OSA patients (SMD = 0.87, *p* < 0.001). By contrast, serum/plasma SOD concentrations were not significantly different between the two groups. Although extreme between-study heterogeneity was observed, the SMD was not substantially modified when individual studies were sequentially removed. In conclusion, we observed that whole blood, but not serum/plasma, SOD concentrations were significantly lower in OSA patients compared with controls. Our meta-analysis suggests an impaired antioxidant defence in OSA that is more robustly assessed in the corpuscular biological matrix and provides useful background information for further studies investigating the association between SOD changes and clinical status in OSA.

## 1. Introduction

**Figure 1:**
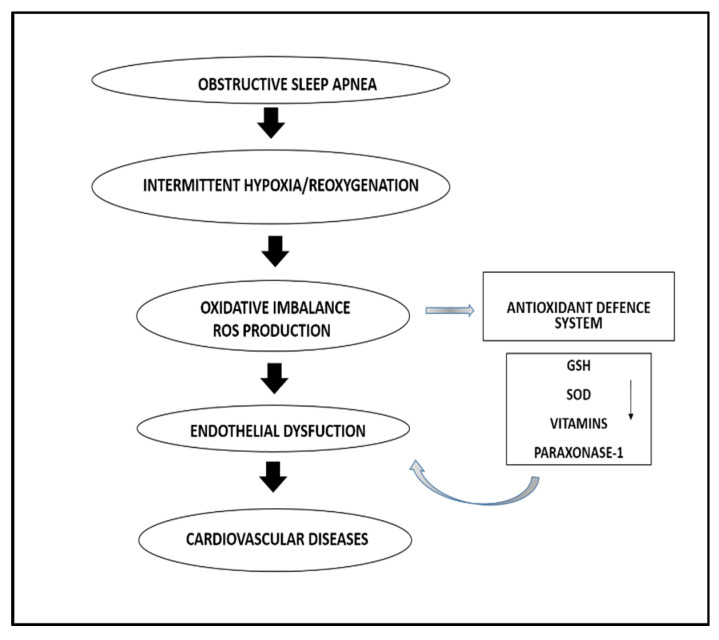
Flow chart describing the role of antioxidant defence molecules in the pathogenesis of OSA. GSH: glutatione; SOD: superoxide dismutase.

Obstructive Sleep Apnoea (OSA) is characterized by episodes of upper airway obstruction and intermittent cycles of desaturation–reoxygenation [1]. In particular, the reoxygenation phase is considered to promote oxidative stress with excessive ROS production, reduced activity of antioxidant defences, or a combination of both [2,3]. Whilst OSA represents an independent risk factor for cardiovascular disease, the exact mechanisms involved in this association are not fully understood [4,5]. The imbalance between ROS production and antioxidant capacity might account, at least in part, for the link between OSA and cardiovascular disease [6,7]. Under conditions of oxidative stress, the superoxide radical, generated principally during mitochondrial respiration, represents the most abundant ROS [8]. This key agent is capable of oxidizing a variety of cellular components including lipids, proteins and DNA, with consequent alteration of their biological functions [9]. The antioxidant system, constituted by different molecules and enzymes, represents a critical defence against radicals-related damage. Superoxide dismutase (SOD) is a metallic enzyme that allows the dismutation of the superoxide anion into molecular oxygen and hydrogen peroxide [8,10]. Among the antioxidant defence mechanisms, SOD is primarily involved in the process of detoxification of superoxide radicals. Thus, a decrease in the circulating concentrations of this enzyme would reflect an impaired antioxidant defence (Figure 1). Several studies have investigated the role of oxidative stress markers, including indicators of lipid peroxidation, protein carbonylation and DNA oxidation, as well as the activity of the antioxidant enzymes SOD and glutathione peroxidase, in OSA [11,12,13]. While these studies generally reported correlations between some of these markers and the presence and severity of OSA, conflicting results exist regarding SOD concentrations in this group [14]. Furthermore, CPAP therapy, a fundamental treatment strategy in OSA also in view of its antioxidant effects, have shown to have little effect on SOD concentrations, in contrast to oxidative stress markers such as 8-isoprostane and 8-hydroxy-2-deoxyguanosine [15]. Therefore, in order to capture and critically appraise the available evidence on the pathophysiological role of SOD, we performed a systematic review and meta-analysis of studies reporting SOD concentrations in serum, plasma, and whole blood in OSA patients and non-OSA controls to detect possible differences between the two groups. 

## 2. Materials and Methods

### 2.1. Search Strategy, Eligibility Criteria, and Study Selection

We conducted a systematic search of publications in the electronic databases Pubmed, Web of Science, Scopus and Google Scholar, from inception to June 2021, using the following terms and their combination: “superoxide dismutase” or “SOD” and “Obstructive Sleep Apnoea Syndrome” or “OSAS” or “OSA” or “OSAHS” (PROSPERO registration number: CRD42021272833). 

In order to establish relevance, the abstracts were screened independently by two investigators. If relevant, the two investigators independently reviewed the full articles. The references of the retrieved articles were also searched to identify additional studies. A third investigator was involved in presence of any disagreement between the reviewers. The eligibility criteria were: (i) evaluation of SOD concentrations in blood, plasma or serum/plasma; (ii) comparison between subjects with OSA and non-OSA (case–control design); (iii) studies reporting apnoea–hypopnea index (AHI) values; (iv) studies in adult patients; (v) sample size ≥10 OSA patients; (vi) English language and (vii) availability of full-text publications. Risk of bias was evaluated using the Joanna Briggs Institute (JBI) Critical Appraisal Checklist for analytical cross-sectional studies, with scores ≥5, 4, and <4 indicating low, moderate, and high risk, respectively [16]. Certainty of evidence was assessed using the Grades of Recommendation, Assessment, Development and Evaluation (GRADE) Working Group system, which considers the study design (randomized vs. observational), risk of bias (JBI checklist), presence of unexplained heterogeneity, indirectness of evidence, imprecision of results (sample size, 95% confidence interval width and threshold crossing), effect size (small, SMD < 0.5, medium, SMD 0.5–0.8, and large, SMD > 0.8), and high probability of publication bias.

### 2.2. Statistical Analysis

Considering that the SOD concentrations were expressed using different units of measurement (U/L, U/gHb, ng/mL or %), the forest plots of continuous data and the assessment of the differences in SOD concentrations in OSA patients vs. non-OSA were expressed using the standardized mean differences (SMDs). The presence of *p* < 0.05 was considered statistically significant, and 95% confidence intervals (CIs) were reported. When required, the values of mean and standard deviation were extrapolated from median and IQR values, or from median and range as previously reported by Wan et al. [17] as described by Hozo et al. [18] or from graphs using the Graph Data Extractor software. 

The Q statistic was used to test heterogeneity of SMD across studies (significance level at *p* < 0.10). Additionally, the I2 statistic, a quantitative measure of inconsistency between studies, was calculated (I2 < 25%, no heterogeneity; I2 between 25% and 50%, moderate heterogeneity; I2 between 50% and 75%, large heterogeneity; and I2 > 75%, extreme heterogeneity) [19,20]. Statistical heterogeneity was established in the presence of an I2 statistic value ≥50% [20]. When the analyses detected high heterogeneity, a random-effects model was applied. Sensitivity analysis was also performed to test the influence of any single study on the overall risk estimate by excluding each study sequentially [21]. For each effect size the confidence intervals at 95% (CIs) and the overall effect were reported, and *p* < 0.05 was considered statistically significant. The study was compliant with the principles outlined in the PRISMA 2020 Statement [22] concerning the reporting of systematic reviews and meta-analyses. The software Stata 14 was used to perform statistical (STATA Corp., College Station, TX, USA).

## 3. Results

### 3.1. Systematic Research

**Figure 2:**
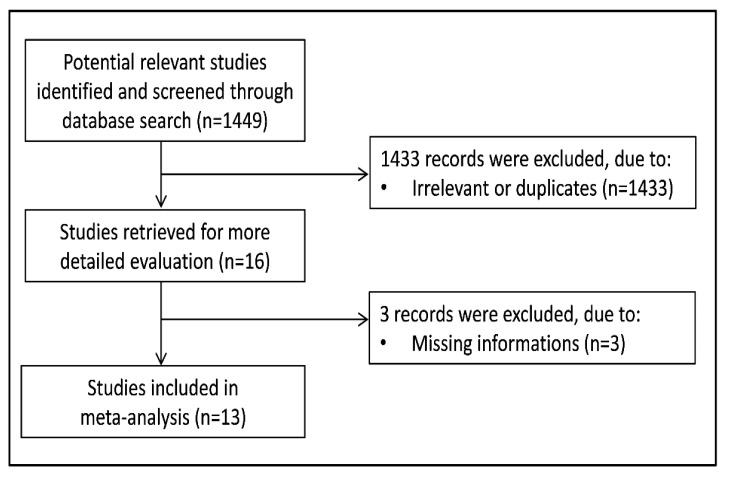
Flow chart of study selection.

A flow chart describing the screening process is presented in Figure 2. We initially identified 1449 studies. A total of 1433 studies were excluded after the first screening because they were either duplicates or irrelevant. After a full-text revision of 16 articles, three studies were further excluded because of missing information. Thus, thirteen studies were included in the meta-analysis [14,23,24,25,26,27,28,29,30,31,32,33,34]. A total of 847 OSA patients (mean age 47 years, 74% males) and 438 controls (mean age 50 years, 72% males) were evaluated. The characteristics of the retrieved studies, published between 2005 and 2020, are presented in Table 1.

### 3.2. Meta-Analysis of Blood SOD Concentrations

#### 3.2.1. Study Characteristics

Eight studies with a total of 256 OSA patients (mean age 49 years, 82% males), and 175 controls (mean age 52 years, 84% males) were evaluated [23,24,25,26,27,28,29,30].

#### 3.2.2. Risk of Bias

The risk of bias was considered low in seven studies [24,25,26,27,28,29,30] and moderate in the remaining one [23] (Table 2).

#### 3.2.3. Results of Individual Studies and Syntheses

**Figure 3:**
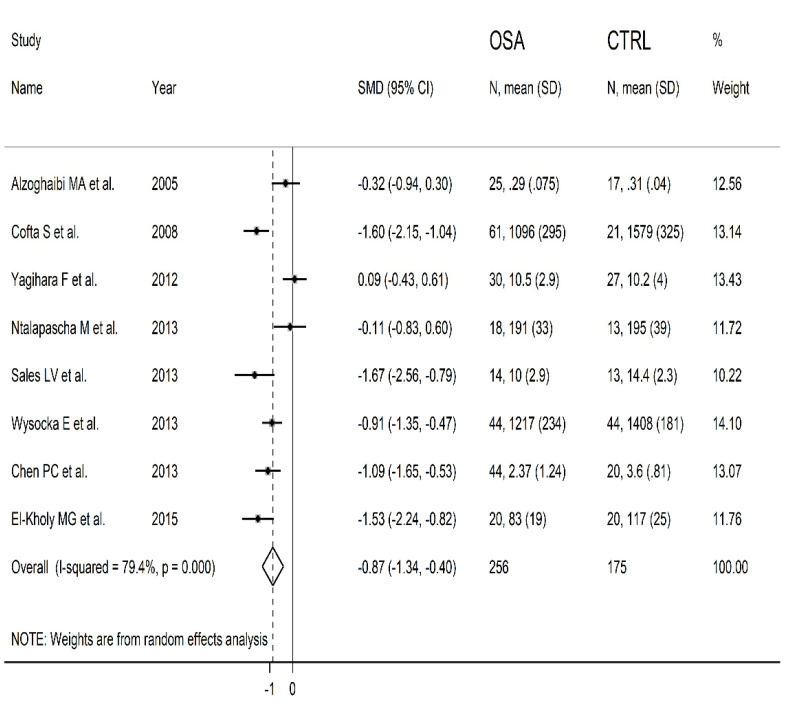
Forest plot of studies examining blood SOD values of OSA and controls.

**Figure 4:**
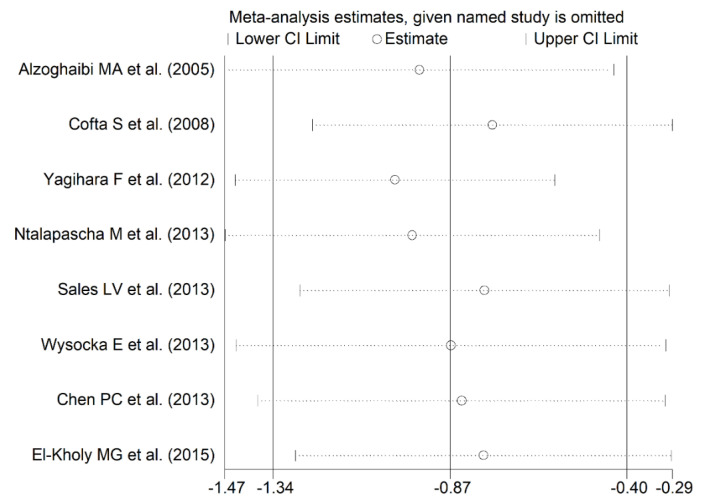
Sensitivity analysis of the association between blood SOD and OSA disease. The influence of individual studies on the overall standardized mean difference (SMD) is shown. The middle vertical axis indicates the overall SMD and the two vertical axes indicate the 95% confidence intervals (CI). Hollow circles represent the pooled SMD when the remaining study is omitted from the meta-analysis. Two ends of each broken line represent 95% CI.

The forest plot for blood SOD concentrations in OSA patients and controls in the eight studies is reported in Figure 3. In seven studies [23,24,26,27,28,29,30], OSA patients had lower blood SOD concentrations compared to controls (mean difference range, −1.67 to −0.11) and the difference was statistically significant in five [24,27,28,29,30]. Extreme heterogeneity between studies was observed (I2 = 79.4%, *p* < 0.001). Thus, random-effects models were used. Overall, pooled results showed that blood SOD concentrations were significantly lower in patients with OSA (SMD = −0.87, 95% CI −1.34 to −0.40; *p* < 0.001). Sensitivity analysis showed that the corresponding pooled SMD values were not substantially altered when any single study was in turn omitted (effect size range, between −0.97 and −0.76, Figure 4).

#### 3.2.4. Publication Bias

Assessment of publication bias was not possible because of the relatively small number of studies.

#### 3.2.5. Certainty of Evidence 

The initial level of certainty for blood SOD SMD values was considered low because of the observational nature of the selected studies (rating 2, ⊕⊕⊝⊝). After considering the presence of a low risk of bias in seven out of eight studies (no rating change required), a generally extreme and unexplained heterogeneity (serious limitation, downgrade one level), lack of indirectness (no rating change required), the relatively low imprecision (relatively narrow confidence intervals without threshold crossing, upgrade one level), the relatively large effect size (SMD −0.87, upgrade one level), and lack of assessment of publication bias (downgrade one level), the overall level of certainty was considered low (rating 2, ⊕⊕⊝⊝).

### 3.3. Meta-Analysis of Serum/Plasma SOD Concentrations

#### 3.3.1. Study Characteristics

Five studies with a total of 591 OSA patients (mean age 46 years, 71% males) and 261 controls (mean age 49 years, 65% males) were evaluated [14,31,32,33,34].

#### 3.3.2. Risk of Bias

The risk of bias was considered low in all studies (Table 2).

#### 3.3.3. Results of Individual Studies and Syntheses

**Figure 5:**
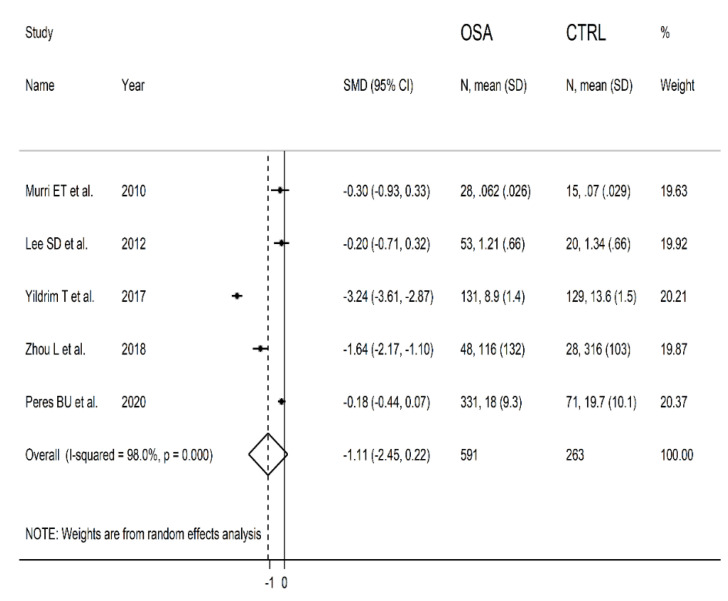
Forest plot of studies examining serum/plasma SOD values of OSA and controls.

**Figure 6:**
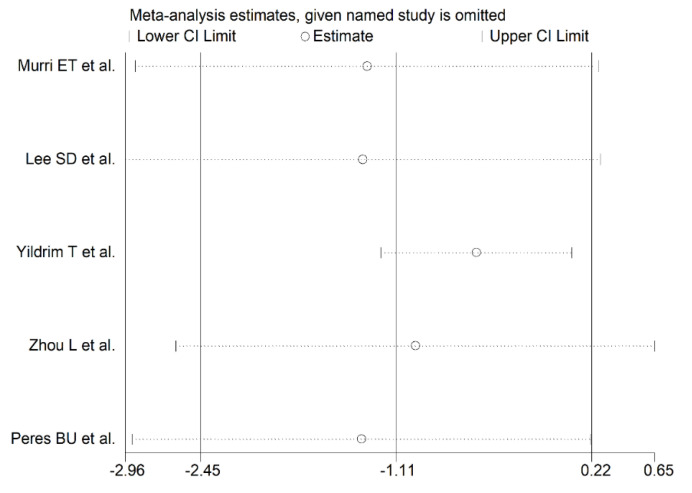
Sensitivity analysis of the association between serum/plasma SOD and OSA disease. The influence of individual studies on the overall standardized mean difference (SMD) is shown. The middle vertical axis indicates the overall SMD and the two vertical axes indicate the 95% confidence intervals (CI). Hollow circles represent the pooled SMD when the remaining study is omitted from the meta-analysis. Two ends of each broken line represent 95% CI.

The forest plot for serum/plasma SOD concentrations in OSA patients and controls in the five studies is reported in Figure 5. In all studies [14,31,32,33,34], OSA patients had lower serum/plasma SOD concentrations compared to controls (mean difference range, −3.24 to −0.18) and the difference was statistically significant in two [32,33]. Extreme heterogeneity between studies was observed (I2 = 98.0%, *p* < 0.001). Thus, random-effects models were used. Overall, pooled results showed that serum/plasma SOD values were non-significantly lower in patients with OSA (SMD = −1.11, 95% CI −2.45 to +0.22; *p* = 0.10). The effect size was not substantially altered (range between −1.35 and −0.57, Figure 6), both in direction and magnitude, after sequentially removing individual studies.

#### 3.3.4. Publication Bias

Assessment of publication bias was not possible because of the relatively small number of studies.

#### 3.3.5. Certainty of Evidence 

The initial level of certainty for serum/plasma SOD SMD values was considered low because of the observational nature of the selected studies (rating 2, ⊕⊕⊝⊝). After considering the presence of a low risk of bias in all studies (no rating change required), a generally extreme and unexplained heterogeneity (serious limitation, downgrade one level), lack of indirectness (no rating change required), the relatively high imprecision (confidence intervals with threshold crossing, downgrade one level), the relatively large effect size (SMD −1.11, upgrade one level), and lack of assessment of publication bias (downgrade one level), the overall level of certainty was considered very low (rating 0, ⊝⊝⊝⊝).

## 4. Discussion

The pro-oxidant state resulting from the recurring hypoxia and reoxygenation cycles appears to be involved in the association between OSA and cardiovascular diseases [35]. Whilst the relationship between oxidative stress and OSA has been extensively explored, controversial results have been reported [36,37]. Several studies investigated the concentrations of oxidant molecules in OSA patients, and their correlation with the severity of the disease [38,39]. A more limited number of studies have sought to elucidate the activity of key antioxidant enzymes, such as SOD and glutathione peroxidase [15,40,41]. SOD represents an important antioxidant mechanism in the detoxification of the superoxide anion, the most abundant ROS molecule [42]. Consequently, the measurement of the circulating concentrations of this enzyme might be useful to monitor the antioxidant defences in OSA patients and the effect of therapies [43]. 

Our systematic review and meta-analysis sought to address the potential of SOD to characterize the presence of oxidative stress in OSA by evaluating the presence and the effect size of differences in the concentrations of this enzyme between OSA patients and controls. In all studies, the concentrations of SOD in were lower in OSA than in non-OSA controls. When assessing specific biological matrices, the overall SMD value of whole blood SOD concentrations was statistically significant whereas the pooled SMD of serum/plasma was not. 

The SOD enzyme is present in three isoforms in humans, with distinct subcellular localization, cytoplasmic, mitochondrial, and extracellular for SOD1, SOD2, and SOD3, respectively. This different location is important for compartmentalized redox signalling [42]. Particularly, the significant reduction of SOD activity in whole blood could be the result of the high exposure of blood cells, particularly the erythrocytes, to oxidative stress [44]. A possible reason for the discrepancy in the results from studies on whole blood vs. those on serum/plasma is that the measured concentrations of SOD in the former may reflect the activity of all three isoforms while those in the latter may specifically reflect the activity of the extracellular SOD3 isoform. 

Whilst extreme between-study heterogeneity was observed both in studies on whole blood and in those on serum/plasma in sensitivity analysis the SMD was not substantially altered when individual studies were in turn omitted. The observed heterogeneity could be explained by a number of unreported factors, including pre-analytical information and patient characteristics such as dietary patterns, medications, smoking status, and comorbidities that might influence the oxidative/antioxidative balance.

A recent meta-analysis of 10 studies reported lower SOD concentrations in OSA when compare with the non OSA-control [45]. However, differently from this study, we conducted separate analyses according to the different biological matrix investigated, plasma/serum vs. whole blood, and we observed significant differences in SOD between OSA and non-OSA groups in whole blood, but not in serum. Furthermore, our analyses were enriched by a comprehensive assessment of the certainty of evidence according to GRADE. Therefore, the two studies provide complementary information on this issue. 

In addition to the high heterogeneity observed, this study has some other limitations mainly due to the relatively small number of studies included and the paucity of information available to assess the presence of publication bias and the relationship between the SMD and disease severity based on the AHI. This prevented the conduct of a meta-regression analysis to investigate associations between the study effect size, study design, and analytical characteristics. These issues are likely to have affected the overall level of certainty that was considered to be low. Furthermore, the number of studies included was limited to those written in English.

Despite these limitations, our study represents the first meta-analysis that evaluated the association between OSA and SOD, according to the different biological matrix used, thus providing a useful background for the design and the conduct of larger prospective studies.

## 5. Conclusions

In conclusion, we observed that OSA patients have significantly lower whole blood concentrations of the antioxidant enzyme SOD in when compared to non-OSA controls. This supports the proposition that OSA is characterized by an impaired antioxidant capacity and highlights the potential utility of assessing SOD concentration in the corpuscular biological matrix. Prospective studies are required to further confirm the validity of SOD assessment in OSA to monitor the progress of the disease and the effect of specific therapies.

## Figures and Tables

**Table 1:** Summary of the studies on controls vs. OSA subjects included in the meta-analysis.

	Control			OSA					
First Author Year, Country	n	Age Mean	Gender (M/F)	SOD Mean ± SD	n	Age Mean	Gender (M/F)	SOD Mean ± SD	AHI Events/h
BLOOD									
Alzoghaibi MA et al., 2005, Saudi Arabia	17	50	NR	0.31 ± 0.04 U/L	25	31	NR	0.29 ± 0.08 U/L	73.5
Cofta S et al., 2008, Poland	21	53	11/10	1579 ± 325 U/gHb	61	52	43/18	1096 ± 295 U/gHb	24
Yagihara F et al., 2012, Brasil	27	66	27/0	10.2 ± 4.0 U/mgHb	30	66	30/0	10.5 ± 2.9 U/mgHb	38
Ntalapascha M et al., 2013, Greece	13	49	13/0	195 ± 39 ng/mL	18	50	18/0	191 ± 33 ng/mL	24
Sales LV et al., 2013, Brasil	13	37	13/0	14.4 ± 2.3 U/mgHb	14	36	14/0	10.0 ± 2.9 U/mgHb	36
Wysocka E et al., 2013, Poland	44	55	44/0	1408 ± 181 U/gHb	44	53	44/0	1217 ± 234 U/gHb	26
Chen P et al., 2013, Taiwan	20	42	15/5	3.60 ± 0.81 U/mgHb	44	42	33/11	2.37 ± 1.24 U/mgHb	15
El-Kholy MG et al., 2015, Egypt	20	49	10/10	117 ± 25 U/mL	20	51	9/11	83 ± 19 U/mL	30
SERUM/PLASMA									
Murri ET et al., 2010, Spain	15	54	15/0	0.070 ± 0.029 U/mL	28	46	NR	0.062 ± 0.026 U/mL	52
Lee SD et al., 2012, South Korea	20	44	20/0	1.34 ± 0.66 U/mL	53	47	53/0	1.21 ± 0.66 U/mL	32
Yildrim T et al., 2017, Turkey	129	49	78/51	13.6 ± 1.5 NR	131	49	74/57	8.9 ± 1.4 NR	34
Zhou L et al., 2018, China	28	45	20/8	316 ± 103 ng/mL	48	41	NR	116 ± 132 ng/mL	47
Peres BU et al., 2020, Canada	71	52	38/33	19.74 ± 10.05 %	331	45	237/94	18.00 ± 9.30 %	26

AHI: apnoea–hypopnea index; SOD: superoxide dismutase; NR = Not reported.

**Table 2:** The Joanna Briggs Institute critical appraisal checklist for analytical cross-sectional studies.

Study	Were the Criteria for Inclusion in the Sample Clearly Defined?	Were the Study Subjects and the Setting Described in Detail?	Was the Exposure Measured in a Valid and Reliable Way?	Were Objective, Standard Criteria Used for Measurement of the Condition?	Were Confounding Factors Identified?	Were Strategies to Deal with Confounding Factors Stated?	Were the Outcomes Measured in a Valid and Reliable Way?	Was Appropriate Statistical Analysis Used?	Risk of Bias
Alzoghaibi	Unclear	Yes	Yes	Yes	No	No	Yes	No	Moderate
Cofta	Yes	Yes	Yes	Yes	No	No	Yes	No	Low
Yagihara	Yes	Yes	Yes	Yes	No	No	Yes	No	Low
Nptalapascha	Yes	Yes	Yes	Yes	Yes	Yes	Yes	Yes	Low
Sales	Yes	Yes	Yes	Yes	No	No	Yes	No	Low
Wysocka	Yes	Yes	Yes	Yes	No	No	Yes	No	Low
Chen	Yes	Yes	Yes	Yes	No	No	Yes	No	Low
El-Kholy	Yes	Yes	Yes	Yes	No	No	Yes	No	Low
Murri	Yes	Yes	Yes	Yes	No	No	Yes	No	Low
Lee	Yes	Yes	Yes	Yes	Yes	Yes	Yes	Yes	Low
Yildirim	Yes	Yes	Yes	Yes	No	No	Yes	No	Low
Zhou	Yes	Yes	Yes	Yes	Yes	Yes	Yes	Yes	Low
Peres	Yes	Yes	Yes	Yes	Yes	Yes	Yes	Yes	Low

## Data Availability

The data presented in this study are available in review.
